# Sodium supplementation has no effect on endurance performance during a cycling time-trial in cool conditions: a randomised cross-over trial

**DOI:** 10.1186/1550-2783-10-30

**Published:** 2013-06-03

**Authors:** Samuel David Cosgrove, Katherine Elizabeth Black

**Affiliations:** 1 Department of Human Nutrition, University of Otago, P.O. Box 56, Dunedin, Otago 9054, New Zealand

**Keywords:** Sodium, Salt, Hyponatremia, Endurance exercise, Hydration, Fluid balance, Thirst

## Abstract

**Background:**

Sodium ingestion during exercise may exert beneficial effects on endurance performance by either its ability to attenuate the decrease in plasma volume or reduce the risk of Exercise Associated Hyponatremia (EAH). This study aimed to investigate the effect of sodium supplements on endurance performance during a 72 km road cycling time-trial in cool conditions (13.8 ± 2.0°C).

**Methods:**

Nine well-trained cyclists (5 male, 4 female) participated in this randomized, double-blinded cross-over study, receiving either a 700 mg^.^h^-1^ salt capsule, or a corn flour placebo during the time trial. Water was ingested ad-libitum throughout the time trial. Measurements were taken pre, post, and 40 min following time-trials, analysing blood, sweat, and urinary hydration and sodium concentration.

**Results:**

Sodium supplements had no effect on time-trial performance (overall time = 171 min sodium vs. 172 min placebo; p = 0.46). There was also no effect on the change in plasma sodium concentration from pre to post time trial between trials (relative plasma [Na^+^] change (pre-post): sodium = 0.56%, placebo = 0.47%; p = 0.60). The greatest difference observed was a significantly change in plasma volume from pre to post exercise between the salt and the placebo trial (p = 0.02), which corresponded with an increased thirst with sodium supplementation.

**Conclusion:**

Sodium supplements therefore do not improving performance during exercise of approximately 3 h duration in cool conditions.

## Introduction

The ingestion of sodium during exercise may be of benefit to performance by maintaining plasma volume [1,2], and/or by attenuating declines in blood sodium, however, at present the influence of sodium ingestion during exercise on performance appears inconclusive [3]. Vrijens and Rehrer [4] showed improved time to exhaustion and attenuated plasma [Na^+^] drops with the ingestion of 61 mmol sodium (18 mmol^.^L^-1^ solution) compared to a placebo drink (distilled water) during 3 h cycling in the heat. Anastaiou and colleagues showed that even small amounts of sodium (19.9 mmol^.^L^-1^; 39.8 mmol in total) ingested during three hours of exercise in the heat were sufficient to attenuate the decrease in plasma sodium relative to water [5]. Similar findings were seen by Twerenbold et al. [6] during a four hour running time trial in temperatures ranging from 5 to 19°C. Again, sodium ingestion (25 mmol^.^h^-1^, 100 mmol total) resulted in a smaller decrease in plasma sodium concentration from pre to post run amongst female athletes. Conversely Barr et al. reported no significant differences in plasma sodium concentration at the end of 6 hours of exercise in the heat when water or a saline solution was ingested, they postulated that the reasons for the lack of difference between the two trials was due to changes in extracellular/intracellular fluid volumes, the incomplete absorption of sodium from the intestine and a greater conservation of sodium within the body during the water trial [7].

Interestingly there were high rates of hyponatremia during the study of Twerenbold et al. study demonstrating that hyponatremia can occur in cold environments when over-drinking is induced this is also highlighted by the mathematical equations of Montain, Cheuvront and Sawka [8]. Despite the positive effects seen in the laboratory these studies employed a fluid intake regime that probably resulted in over-drinking or do not reflect the practices of athletes. Fluid strategies have either ingested fluids to match sweat losses or drinking at a rate to increase body mass over the exercise period. Such drinking practices are not recommended [9] and are known to increase the risk of EAH, therefore these studies lack some application to the real world setting, where athletes generally ingest around 50% of their sweat losses [9].

Two studies have investigated sodium supplementation during ironman races [10,11] both reported no performance differences between those taking sodium supplements and those without sodium during ironman triathlons. However, the controls on the sodium intake of the control group were minimal and the design of the study meant that the numerous factors other than sodium which are known to influence performance were not controlled i.e. training load, carbohydrate intake, genetic physiology. Therefore the effects of sodium supplementation during exercise with ad-libitum fluid intake whilst controlling other factors during exercise are still unclear.

This study aimed to build on the previous evidence, and investigate whether oral sodium supplementation during exercise improves performance during a 72 km cycling time-trial. It was hypothesized that sodium supplements would attenuate the decline in plasma [Na^+^] and plasma volume during the time-trial, and thus improve time-trial performance. As the aetiology of EAH is also closely related to hydration during exercise, a secondary aim was to investigate fluid balance variables in response to supplementation.

## Methods

### Subjects

Nine healthy and well-trained cyclists (5 men, 4 women, mean age 26.8 ± 9 yr, VO_2_max 61.9 ± 7.7 mL^.^kg^-1.^min^-1^) completed both experimental time-trials, which was previously approved by the University of Otago Human Ethics Committee (Dunedin, Otago, New Zealand) and complied with the Helsinki Declaration. Each participant provided written informed consent prior to beginning the study.

### Study design

#### Data collection

Participants completed a double-blinded randomised crossover study, consisting of a pre-testing session, familiarisation trial, and two experimental time trials separated by 7 – 14 days during which time participants were asked to do minimal training. The pre-testing session involved a graded VO_2_max test on a stationary cycle ergometer (Monark 915E, Varberg, Sweden), with gaseous exchange measured on a Metalyser 3B (Cortex, Biophysik GmbH, Leipzig, Germany). The test began with a 5 min warm-up at a light intensity. Workload then increased every 3 min, with heart rate (Polar 310, Polar, Oulu, Finland) and Rating of Perceived Exertion (RPE) on the Borg scale [12] measured in the last 30 s of each stage. VO_2_max was determined when heart rate was consistently within 10 beats of the calculated maximum, the RPE exceeded 19 on the Borg scale [12], the participant was unable to maintain an RPM above 70 rpm, or the RER was consistently above 1.10. A level 1 trained International Society of Advanced Kinanthropometry (ISAK) anthropometrist also performed an anthropometric assessment during this initial visit, collecting a ‘restricted profile’ as described by ISAK [13]. The ‘restricted profile’ includes a sum of 8 skinfolds, waist and hip girth, body mass, and height.

The familiarisation and experimental time-trials were a 72 km ‘out and back’ road cycle over hilly terrain in Dunedin, New Zealand, in which participants used their own road cycle. Pre-trial diets were replicated from a one day estimated diet record kept in the day preceding the familiarisation trial. Likewise, participants arrived to all trials fasting, and a standardised pre-race breakfast (3152 ± 1847 kJ; 27 ± 11 g protein; 112 ± 49 g CHO; 11 ± 12 g total fat) was provided to participants one hour before the time-trial started.

Measurements took place immediately pre and post time-trial, and then once more after a post-race meal approximately 40 min from finishing, all samples were obtained in the sitting position. A 1 mL capillary blood sample was collected after appropriate cleaning with an alcohol swab, via fingerprick, (Unistick 3 extra lancet, Owen Mumford, Oxford, United Kingdom) and analysed using an i-STAT point of care analyser with a CG8+ cartridge (Abbott Point of Care Inc, Illinois, USA). This provides measures of sodium, haematocrit, and haemoglobin from these measures plasma volume was calculated using the equations of Dill and Costill [14]. Participants were then asked to provide a urine sample in private, which was collected in a 20 mL sealed, sterile plastic tube (Techno Plas, South Australia, Australia) and stored at 4°C until laboratory analysis. A 100 mm visual analogue scale subjective questionnaire regarding thirst, gastrointestinal distress, as previously utilised by Rolls et al. [15] was also completed by participants both pre and post time-trial. Body mass was measured on electronic scales to the nearest 0.1 kg (Tanita-Wedderburn TBF-310, Illinois, USA) in minimal clothing. Finally, sweat patches (Tagaderm patch + pad, 3 M, Loughborough, UK) were applied to the upper back, forearm, chest and mid thigh on the right-hand side of the body which was first cleaned with deionised water and dried. The patches remained in place throughout the trial. Immediately following the time-trial the patches were removed with sterile tweezers and stored in a 30 mL sealed, sterile plastic tube (Techno plas, South Australia, Australia) at 4°C.

The time-trial course was on a sheltered, this limited the exposure to the wind which was also minimised by starting the time-trials early in the morning a time when wind is minimal, but hilly cycle route in Dunedin, New Zealand, with a total of 1 556 m in elevation gained in the 72 km. Cyclists were given a coded, clear zip-lock bag each containing 15 clear capsules with either 233 mg sodium chloride, or an identical corn flour placebo. Participants were instructed to consume three capsules for every hour, which equated to 700 mg NaCl^.^h^-1^, consistent with doses used in previous trials [2,11], and recommended by Zapf et al. [16]. Water and ‘Jet Plane’ lollies (Pascall, Auckland, New Zealand) could be consumed ad libitum during the trial but the weights consumed were recorded to the nearest 0.1 g (Salter Vista Electronic Scales, England). Participants were fitted with a Global Positioning System (GPS) stopwatch and heart rate monitor (Garmin Forerunner 110, Garmin, Kansas, USA) for the time-trial. Outside air temperature, humidity, and weather were recorded every 15 min during the time-trials using a WS9623 Wireless 868 MHz Weather Station (La Crosse Technology, France).

#### Data analyses

Performance was assessed via overall time to complete the time-trial. The cyclists’ uphill time splits were also used as a measure of performance to account for any variation in skill in descending the hills. Plasma [Na^+^] (mmol^.^L^-1^), haematocrit, and blood glucose values (mmol^.^L^-1^) were analysed via the i-STAT point of care analyser (Abbott Point of Care Inc, Illinois, USA) and recorded in the field. Sweat sodium and chloride concentration (sweat [Na^+^], sweat [Cl^-^]) was analysed in small batches through a Cobas C311 module (Roche Diagnostics, Basel, Switzerland) using the Ion Selective Electrode (ISE) technique (mean CV = 2.01 ± 1.59%). Sweat sodium concentrations were then extrapolated to whole body sweat sodium losses using the calculations of Patterson et al. [17]. To ensure contamination of the patches nor leaching from the skin had not occurred sweat potassium was measured and all samples were within the normal range [18]. Urine osmolality was measured via freezing point depression (Osomat 030, Genotec GmbH, Baden-Wurttemberg, Germany), to indicate hydration status. Subjective feelings of thirst were indicated on a 100 mm visual analogue scale, which was used as a rating from 0 (not thirsty at all) to 100 (extremely thirsty) [15].

### Statistical analysis

Statistical analyses were performed using Stata Version 11.2 (StataCorp, Texas, USA). Normality of the data was evaluated using a Shapiro-Wilks test, and difference in variance was assessed by two-group variance comparison tests before all comparisons.

Multivariate regression was used to assess the effect of sodium supplements on exercise performance and plasma [Na^+^]. Differences in overall time and uphill time were compared whilst controlling for temperature and weather (wet or dry road). The difference in absolute (mmol^.^L^-1^) and relative (%) plasma [Na^+^] change was analysed controlling for average heart-rate. A paired t-test was also used to investigate differences in plasma [Na^+^] from pre-race to post-race within each intervention.

Urine and sweat concentrations were well distributed and the absolute (mmol^.^L^-1^) and relative (%) change in electrolytes in each were analysed using a Student’s t-test. Changes in body mass, haematocrit, plasma volume change and fluid intake were assessed using multivariate regression controlling for mean heart rate and temperature.

Statistical significance was set at p ≤ 0.05. If a relationship was close to statistical significance, a Cohen’s *d* effect size was also calculated. Data is reported as mean ± standard deviation (SD).

## Results

Descriptive characteristics of the participants are shown in Table 1. Participants were lean, with a mean sum of eight skinfolds of 82.9 mm, which equates to a mean body fat percentage of 8.1 ± 0.71% for males and 16.2 ± 1.3% for females using the Yuhasz equation [19].

**Table 1:** Physical characteristics

	**Participants (n = 9)**	**Males (n = 5)**	**Females (n = 4)**
Age (years)	26.8 ± 9.0	25.0 ± 5.4	29.8 ± 13.1
Height (cm)	175.1 ± 9.74	182.4 ± 5.8	166.9 ± 3.78
Weight (kg)	72.8 ± 12.2	80.0 ± 11.4	63.8 ± 5.7
BMI (kg/m^2^) ^a^	23.6 ± 2.1	24.0 ± 2.4	23.2 ± 1.5
VO_2_max (L^.^min^-1^)	4.5 ± 0.98	5.2 ± 0.72	3.7 ± 0.44
VO_2_max (mL^.^kg^-1^.min^-1^)	61.9 ± 7.7	65.0 ± 4.5	57.9 ± 9.3

^a^ body mass index.

Age (years), Height (cm), Weight (kg), BMI (kg/m^2^), and VO_2_max for the male and female participants separately and combined.

Environmental conditions during the trials were mildly cold. Mean temperatures during the trials were not different between the sodium and placebo interventions, with temperatures of 14.0 ± 2.1°C and 13.5 ± 2.1°C respectively (p = 0.70). Likewise, mean humidity (63.1 ± 9.8%) was not different between the interventions (p = 0.52). The proportion of trials completed on a wet road was also similar between the sodium and placebo trials, 33% vs. 56% respectively (p = 0.34). There was no significant difference in performance between the wet road and dry road trials (p = 0.17).

### Athletic performance

Overall time to finish was not different between interventions, being 172.3 ±23.3 min and 171.3 ± 23.5 min in the placebo and sodium trials respectively (p = 0.46)(Table 2). The fastest time to complete the course was 153.2 min in the sodium trial and 154.4 min in the placebo trial. Six participants were faster with the sodium supplementation compared to three with the placebo. The uphill time splits between the sodium and placebo interventions were also not different, with the placebo and sodium times being 118.4 ± 18.4 min and 118.7 ± 19.0 min respectively (p = 0.98).

**Table 2:** Performance variables

**Performance variables**	**Placebo**	**Sodium**	**P**
Overall time (min)	172.3 ± 23.3	171.3 ± 23.5	0.46
Uphill time (min)	118.4 ± 18.4	118.7 ± 19.0	0.98
Mean heart rate (beats^.^min^-1^)	157.1 ± 9.2	158.0 ± 9.2	0.86

Mean ± SD performance variables overall time (min), uphill time (min) and heart rate (beats^.^min^-1^) among participants when consuming sodium supplements and placebo.

### Plasma sodium

Pre-race plasma sodium values were significantly higher among those in the sodium intervention compared to the placebo intervention (141.6 ± 1.8 vs. 140.0 ± 1.2 mmol^.^L^-1^, p = 0.047), although both values were within the normal reference range (135 – 145 mmol^.^L^-1^). In contrast to pre-race values, plasma [Na^+^] at the finish of the time-trial (post-race) was not different between the placebo and sodium interventions (P = 0.17). There was no significant change in plasma [Na^+^] from pre-race to post-race in either intervention, the relative change being 0.47 ± 0.02% with the placebo and 0.56 ± 0.02% with sodium (p = 0.7). Following the post-race meal, plasma [Na^+^] was significantly higher among riders in the sodium intervention compared to the placebo intervention (P = 0.02), although this was still within the normal reference range.

### Sweat indices

Sweat rate (placebo, 0.71 ± 0.29 L^.^h^-1^; sodium, 0.55 ±0.22 L^.^h^-1^; P = 0.19) and sweat sodium concentration (placebo, 34.0 ± 14.2 mmol^.^L^-1^; sodium, 37.3 ± 16.2 mmol^.^L^-1^; P = 0.70) were not different between the interventions (Table 3). Consequently, there was no significant difference observed in sweat sodium loss (placebo, 25.3 ± 16.8 mmol^.^h^-1^; sodium, 26.3 ± 16.2 mmol^.^h^-1^; P = 0.29), although the Cohen’s *d* effect size of this comparison is 0.59, indicating a medium effect of the sodium group having higher sweat [Na^+^] losses. Sweat chloride concentration was not different between interventions (P = 0.68).

**Table 3:** Sweat losses and electrolyte concentrations

	**Placebo**	**Sodium**	**P**
Sweat rate (L^.^hr^-1^)	0.71 ± 0.29	0.57 ± 0.22	0.25
Sweat [Na^+^] (mmol^.^L^-1^)	34.0 ±14.2	37.3 ± 16.2	0.70
Sweat sodium loss (mmol^.^h^-1^)	25.3 ± 16.8	26.3 ± 16.2	0.29
Sweat [Cl^-^] (mmol^.^L^-1^)	43.5 ± 18.2	39.5 ± 21.9	0.68

Mean ± SD sweat rate (L^.^h^-1^), sweat sodium concentration (mmol^.^L^-1^), sweat sodium loss (mmol^.^h^-1^), and sweat chloride concentration (mmol^.^L^-1^) among participants when consuming sodium supplements and placebo.

### Fluid balance

Athletes began the time-trial equally hydrated in both trials, according to their pre-race urine osmolality (P = 0.91) (Table 4). This hydration status did not change across the time-trial, and the relative change in urine osmolality from pre-race to post-race was not different between interventions (P = 0.43). No participant urinated during either of the time trials. Participants in both the placebo and sodium intervention lost a mean of 1% body mass over the course of the time trial, from pre-race to post-race. This relative change in body mass was not different between the two interventions (P = 0.52).

**Table 4:** Measures of fluid balance

	**Placebo**	**Sodium**	**P**
Relative body mass change (%)	−1.04 ± 0.55	−0.99 ± 0.80	0.52
Relative plasma volume change (%)	−0.85 ± 1.83	1.78 ± 2.23	0.02*
Pre-race urine osmolality (mosmol^.^L^-1^)	509.9 ± 295.2	493.7 ± 263.7	0.91
Relative urine osmolality change (%)	31.5 ± 121.7	−6.1 ± 43.6	0.43
Fluid intake rate (mL^.^h^-1^)	268.9 ± 65.0	428.42 ± 166.3	0.01*
Thirst change^a^	−0.6 ± 34.2	20.0 ± 23.0	0.17

^a^post-pre, difference in subjective score out of 100; * P < 0.05.

Mean ± SD fluid balance variables: absolute (kg) and relative (%) body mass change, absolute (mL) and relative (mL^.^h^-1^) fluid intake, relative (%) hamatocrit change and pre-trial urine osmolality (mOsmol^.^kg^-1^) among athletes consuming sodium supplements and placebo.

Whilst the absolute haematocrit values at pre-race were similar between the interventions, the changes in these values across the time-trial were different. Haematocrit significantly reduced during the sodium intervention by 3% (P = 0.02), which was significantly different from the observed change in the placebo group, which increased by 1.5% (P = 0.02). The difference in haematocrit is reflected in the significant differences in relative plasma volume change, which increased in the sodium intervention, but decreased in the placebo intervention (sodium = 1.78 ± 2.23%; placebo = −0.85 ± 1.83%; P = 0.02). Fluid intake was also different between the interventions. The sodium group consumed 160 mL^.^h^-1^ more than the placebo group (P = 0.01), resulting in an overall consumption of 430 mL more in sodium intervention over the time-trial. Whilst there was no significant difference in the change in thirst rating (P = 0.17), the sodium group tended to become thirstier during the time-trial (Cohen’s *d* effect size = 0.70).

## Discussion

The findings of this study do not support the premise that sodium supplementation improves endurance performance or affects plasma [Na^+^] in cool conditions. However, there were considerable differences in fluid balance and plasma volume shifts, as well as the novel finding of behavioural changes, such as increased fluid intake.

### Performance

Sodium supplementation had no effect on performance during a cycling time-trial of approximately three hours duration in cool conditions. This disagrees with some laboratory controlled studies [4,5], and the research on pre-exercise sodium loading protocols [20,21] which have shown that volumes of sodium similar to the amounts ingested in this study improve performance. However, the results of this study are consistent with the more recent research using a time-trial or racing situation to assess performance in the field [6,10,11]. The time-trial exercise prescription used in this study was of a similar duration to marathons, triathlons, and many cycling road races; events with reported cases of hyponatremia and targeted guidelines for sodium and fluid intakes [9]. The performance results therefore tend to be more applicable to athletes and coaches, particularly as athletes were able to perform the test at an intensity that reflects their pacing strategies during the race, consistent with the methods of Speedy et al. [11] and Hew-Butler et al. [10].

It is interesting that sodium ingestion before exercise appears to improve performance but the evidence for sodium supplementation during exercise is less clear [20,21]. Pre-exercise sodium loading protocols have generally employed a similar amount of sodium to be ingested in a shorter timeframe and with larger fluid volumes than the present study [20,21]. Even in studies where dehydration has occurred during exercise the initial rate of fluid ingestion was higher than in the present study [22]. This has resulted in a greater difference in plasma volume between the sodium and no sodium trials at the start of exercise compared to the present study [23]. During the present study participants ingested approximately 50% of their sweat losses, and a smaller expansion in plasma volume was seen.

Future studies may wish to investigate the effects of sodium ingestion pre-exercise compared to ingestion during exercise as differences in study protocols, including the volume of fluid ingested, the amount of sodium consumed, participants training status, exercise sessions and the environmental conditions make it difficult to compare the results of study utilising a pre-exercise sodium loading protocol with those investigating sodium supplementation during exercise.

Further the results of this study showed large variability in the change in plasma volume from pre- to post- exercise, so the effects of sodium supplementation maybe more pronounced in some individuals, potentially due to differences in training status or regular dietary sodium intakes. Indeed six of the participants did perform better on the sodium trail, although there was no statistical significant difference in performance in this study. Therefore the results may suggest that some individuals respond to sodium ingestion during exercise whilst others do not, this may be due to differences in training status, sweat sodium losses or renal handling of sodium.

### Plasma sodium concentration

Plasma sodium was significantly greater among the sodium group compared to the placebo group before the time-trial started. Sodium intakes demonstrate considerable day-to-day variation both between and within individuals [24], making dietary manipulation extremely difficult. Such a chronic dietary manipulation would have significantly increased participant burden and may have affected sodium balance during the time-trial. Indeed, whilst the pre-race plasma [Na^+^] values were statistically different between the groups, this difference was small (1.6 mmol^.^L^-1^), and both groups were within the normal reference range. Pre-race plasma [Na^+^] had little effect on the change of plasma [Na^+^] during the time-trial, which remained the same in both groups.

In line with the findings of Barr et al. [7], similar plasma [Na^+^] levels were seen between the trials immediately following exercise (post-race), regardless of whether the participants received a sodium supplement or not, suggest that during an exercise session of this duration, sodium supplementation has little effect on plasma sodium concentrations. However, as all participants remained in the normal reference range of plasma [Na^+^], with no athletes developing hyponatremia, the lowest plasma [Na^+^] value being 137 mmol^.^L^-1^, which occurred during the placebo trial. Whether sodium supplementation would be beneficial in situations where the risk of EAH is greater can not be resolved by this study. Much like previous field studies which found no change in plasma [Na^+^] during an Ironman Triathlon [10,11], the athletes in this study were free to consume fluids ad libitum. This protocol differs from laboratory studies that often had athletes consuming fluid equal to sweat rate [4-6], which some have suggested is over-drinking and possibly not reflective of the majority of athletes’ intake during exercise [10]. The athletes in this study consumed fluid on average just above half their sweat rate, which is consistent with ad libitum fluid intake rates expressed by the American College of Sports Medicine [9]. This resulted in a small decrease in body mass, and is probably the reason for the small but non-significant increase in plasma sodium over the race in both interventions. Considering laboratory studies observed a greater change in plasma [Na+] and higher rates of EAH [4-6], this study adds to the accumulating evidence from field trials that consuming fluid ad libitum during exercise is the most effective means of controlling plasma [Na^+^], irrespective of consuming sodium supplements.

However, the outdoor environment must be considered as a limiting factor when interpreting these results. Whilst the participants’ mean sweat [Na^+^] was within the normal range, the sweat rates observed in this study were considerably lower than endurance races observed in previous observation studies [25-27], thus sodium losses in this study would likely be smaller. The low sweat rates would mean even small fluid intakes could result in overdrinking and potentially result in declines in plasma [Na+] as demonstrated by the calculations of Montain and collegues [8]. Indeed EAH has been reported during events undertaken in 9-12°C [28]. However, as no incidence of hyponatremia was seen amongst the placebo group, it can not be concluded that sodium supplements reduce the incidence of hyponatremia.

### Fluid balance

The increase in plasma volume whilst consuming the sodium supplement, compared to a slight decrease when consuming the placebo, helps to explain the lack of effect on plasma [Na^+^]. Sanders et al. [2] reported similar plasma volume changes in their cross-over intervention study, and explained this difference is due to a fluid shift from the intracellular fluid (ICF) to the extracellular fluid (ECF) when salt tablets are consumed, thus plasma [Na^+^] and osmolality is preserved within normal reference limits, but plasma volume is expanded. Previous research has suggested that the expansion of plasma volume may improve exercise performance [21]. However, if this is at the expense of the intracellular fluid then it is also possible that performance may be impaired as cellular volume plays an important role in muscular metabolism [3,29,30]. Unfortunately, intracellular fluid volume (ICF) was not measured so the effects of sodium ingestion on ICF can not be evaluated. However, in the present study this larger plasma volume had no effect on performance, it did cause significant behavioural changes during exercise, demonstrated by the difference in thirst and fluid intake. Unfortunately, intracellular fluid volume (ICF) was not measured so the effects of sodium ingestion on ICF can not be evaluated.

Despite never actually tasting salt, those in the sodium group tended to become thirstier during the time-trial compared to the placebo group, and consumed 160 mL^.^h^-1^ of additional fluid when consuming sodium supplements. This is likely due to cerebral osmoreceptors being stimulated by the fluid shift from the ICF to the ECF, signalling the hypothalamus to induce thirst [31]. Current guidelines from the American College of Sports Medicine recommend marathon runners drink ad libitum from 0.4 – 0.8 L^.^h^-1^, with consideration of running speed, body weight, and environment [9]. Our results suggest that sodium supplements will affect an athlete’s ad libitum fluid intake by almost 0.2 L^.^h^-1^ during a similar exercise duration. This additional fluid consumption will add weight to elite athletes who aim to maximise a power-to-weight ratio during competition, with no additional performance benefit. This has not been investigated, but it is reasonable to conclude the effect of increased thirst among athletes consuming sodium supplements provides no benefit in a cool environment.

Although not statistically significant it is interesting to note the 0.2 L^.^h^-1^ lower sweat rate with the sodium supplementation this is in line with previous studies [21,22]. This merits further investigation with larger sample size to determine if sodium supplementation negatively effects thermoregulation by increasing plasma osmolality and thus reducing sweat rate and increasing core temperature.

### Limitations

As temperature influences both sweat rates and fluid intakes, which in turn could affect blood sodium concentrations, the cold temperatures in the present study were not ideal. However, between trials there was little difference in the temperature or relative humidity and thus we are able to show the effects of sodium supplementation in mildly cold environments. Future research should investigate the effects of sodium ingestion during exercise in the heat.

## Conclusion

Sodium supplementation had no effect on performance or plasma [Na^+^] during a 72 km cycling time-trial in mildly-cold conditions, however it did appear to influence fluid intake. Well-designed cross-over studies in conditions that would induce larger sweat sodium losses would add constructive evidence in order to provide some practical recommendations for sodium supplementation during endurance sport.

## Competing interests

The authors declare that they have no competing interests.

## Authors’ contributions

KB was responsible for the concept of this project. SC and KB were responsible for the study design, acquisition of data, analysis and interpretation of the data. Both authors were involved with the writing, editing and approval of the final manuscript.
